# Relationship between postoperative nodal skip metastasis of mid-thoracic esophageal squamous cell carcinoma and patient prognosis and its value in guiding postoperative adjuvant treatment

**DOI:** 10.3389/fsurg.2022.1038731

**Published:** 2023-01-09

**Authors:** Hong-Mei Gao, Xiao-Han Zhao, Wen-Bin Shen, You-Mei Li, Shu-Guang Li, Shu-Chai Zhu

**Affiliations:** ^1^ Department of Radiation, Shijiazhuang People’s Hospital, Shijiazhuang, China; ^2^ Department of Radiation Oncology, The Forth Hospital of Hebei Medical University, Shijiazhuang, China

**Keywords:** esophageal neoplasms/esophageal cancer, mid-thoracic, squamous cell carcinoma, nodal skip metastasis, adjuvant treatment, prognosis

## Abstract

**Objective:**

To evaluate the predictive role of nodal skip metastasis (NSM) in the prognosis of lymph node-positive mid-thoracic esophageal squamous cell carcinoma, and to evaluate the significance of postoperative adjuvant treatment in patients with different sites of metastatic nodes.

**Methods:**

A retrospective analysis was performed on clinical data of 321 lymph node-positive mid-thoracic esophageal squamous cell carcinoma patients who underwent surgery in the Fourth Hospital of Hebei Medical University. Based on the site and condition of lymph node metastasis by postoperative pathology, the patients were divided into two groups: NSM group and non-NSM (NNSM) group. The propensity score matching (PSM) method was employed to match the two groups. The prognostic factors of patients before and after PSM as well as the effect of different adjuvant treatment modes on the prognosis of patients before and after PSM were analyzed. SPSS 29.0 statistical software was used for analysis.

**Results:**

PSM in a 1 : 1 matching ratio was performed, 103 patients were assigned to NSM group and NNSM group respectively. Significant differences were found in the 3- and 5-year OS and DFS between the two groups before PSM, the 3- and 5-year OS also showed a significant difference after PSM (*P *< 0.05). Multivariate analysis illustrated that gender, postoperative adjuvant treatment mode, N stage and lymph node metastasis were independent risk factors for OS and DFS after PSM (*P *< 0.05); for NSM patients, postoperative adjuvant chemotherapy and radiotherapy significantly prolonged OS and DFS before and after PSM (*P *< 0.05). But no significant difference was found in OS and DFS for NNSM patients after PSM (*P *> 0.05).

**Conclusion:**

Postoperative NSM is a good prognostic factor for patients with mid-thoracic esophageal squamous cell carcinoma, postoperative adjuvant chemoradiotherapy was recommended for those group, thereby gaining survival benefits.

## Introduction

Esophageal cancer is one of the most common malignant tumors of the digestive tract worldwide (1), whose mortality rate is higher than morbidity rate, regarded as one of the most refractory malignancies (2). According to its anatomical location, the incidence of mid-thoracic esophageal cancer is the highest, accounting for approximately 60% of all esophageal cancer patients (3). Due to the abundant longitudinal lymphatic networks and fewer transverse lymphatic networks in the esophagus, patients with esophageal cancer have a higher rate of nodal skip metastasis (NSM) in mid-thoracic esophagus compared with other sites (4, 5). NSM has demonstrated a favorable prognostic effect in some solid tumors (6, 7). In recent years, more research has focused on NSM in esophageal cancer, however, the results of its prognostic value in patients undergoing esophagectomy remain controversial (4, 8, 9). Therefore, it is urgent to analyze the effect of NSM on the postoperative prognosis of patients with esophageal cancer. In addition, postoperative adjuvant treatment still remains one of the most common treatment approaches for patients undergoing esophagectomy in China, which has a significance prognostic effect, especially for lymph node-positive esophageal cancer patients after surgery (10, 11). However, due to the lack of robust evidence from perspective evidence-based medicine, currently, there has been no consensus on the value of postoperative adjuvant treatment in patients undergoing esophagectomy. In order to further clarify the prognostic value of NSM in patients with thoracic esophageal cancer after esophagectomy, and to analyze the impact of different postoperative adjuvant treatments on the prognosis of patients with different sites of metastatic nodes, we analyzed clinical and pathological data of 321 consecutive patients with positive lymph nodes undergoing surgery for mid-thoracic esophageal squamous cell carcinoma (MT-ESCC).

## Materials and methods

**Figure 1:**
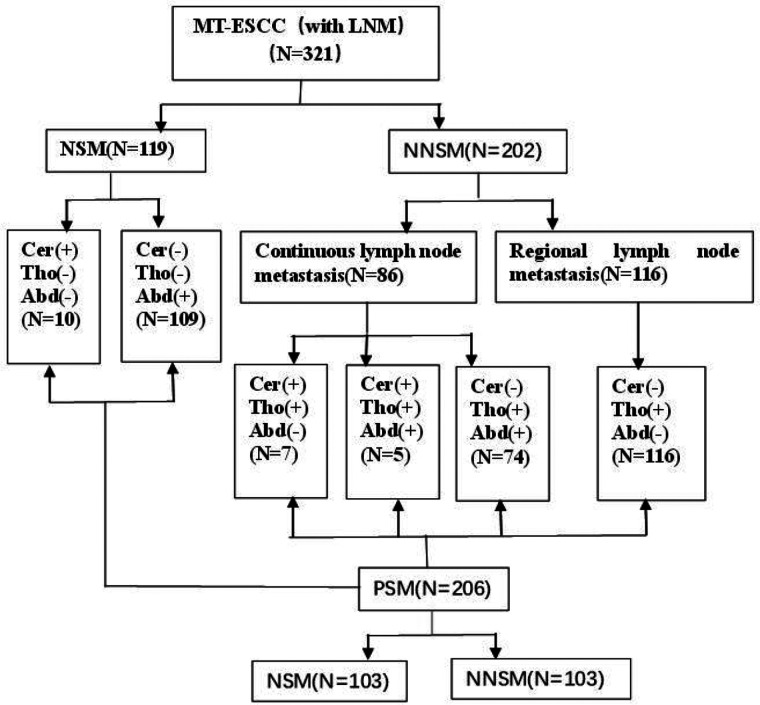
Overview of patient selection and grouping. Note: Cer: cervical; Tho: thoracic; Abd: abdominal; LNM: lymph node metastasis; MT-ESCC: mid-thoracic esophageal squamous cell cancer; NSM: nodal skip metastasis; NNSM: no-nodal skip metastasis; PSM: propensity score matching.

(1) Inclusion criteria: Patients undergoing radical surgery for esophageal cancer at the Fourth Hospital of Hebei Medical University; pathologically confirmed node-positive squamous cell carcinoma; patients with stage pT0-4bN1-3M0 MT-ESCC, according to the 8th edition of the American Joint Committee on Cancer (AJCC) staging system for esophageal cancer (12); no neoadjuvant therapy before surgery; KPS ≥ 70. The exclusion criteria mainly included patients who died during the perioperative period; those with second primary malignancy; the number of lymph nodes dissected <12; and patients with incomplete medical records and follow-up data. The study was conducted in accordance with the Declaration of Helsinki (as revised in 2013). The study was approved by the Ethics Committee of the the Forth hospital of Hebei Medical University and Institute. Individual consent for this retrospective analysis was waived.
(2) Clinical medical records: In total, 321 consecutive MT-ESCC patients who experienced lymph node metastasis after surgery in our hospital from January 2013 to December 2015 and met the inclusion criteria were enrolled in this study. All patients were grouped according to the site of metastatic nodes (Figure 1).
(3) Surgical methods: Before 2015, the surgical treatments for esophageal cancer in Department of Thoracic Surgery of our hospital practically involved oesophagectomy and two-field lymphadenectomy *via* left thoracotomy (Sweet surgery). Afterwards, the Sweet surgery was replaced by minimally invasive McKeown procedure combined with three-field lymphadenectomy as well as Ivor-Lewis surgery combined with two-field lymphadenectomy. In this study, 183 patients underwent Sweet surgery, and 46 and 92 patients underwent minimally invasive McKeown procedure and Ivor-Lewis surgery, respectively.
(4) Definition of the site of metastatic nodes: Lymph nodes were divided into three regions: cervical, thoracic mediastinal, and abdominal regions based on the Japan Esophageal Society (JES) criterion (13). NSM was defined as lymph node metastasis in the abdominal or supraclavicular region, and no metastasis in the thoracic mediastinal region; no-nodal skip metastasis (NNSM) was defined as lymph node metastasis in the thoracic mediastinum region and/or in the abdominal and cervical region (Figure 1).
(5) Postoperative adjuvant treatment mode: Postoperative adjuvant treatment modes in this study was selected primarily based on surgical pathology, oncologists or the hospital where the patients were treated. There were 104, 129 and 88 patients who receive non-adjuvant treatment, postoperative chemotherapy (POCT) and postoperative radiotherapy and chemotherapy (POCRT), respectively. The interval between postoperative radiotherapy and surgery was 3–6 weeks, and the interval between the first cycle of chemotherapy and surgery was 2–4 weeks.

The target area of radiotherapy was mainly the postoperative tumor bed and the corresponding lymph node drainage area of different lesions. The prescribed dose was 45–50.4 Gy/25–28 times, 5 times per week and 1.8–2.0 Gy/time, and all were treated with intensity-modulated radiation therapy. Chemotherapy regimens were mainly platinum-based chemotherapy combined with paclitaxel or 5-fluorouracil. Chemotherapy was conducted for 4–6 cycles, with a median of 4 cycles for patients undergoing chemotherapy alone, and 3–6 cycles with a median of 4 cycles for those undergoing chemotherapy combined radiotherapy.

(6) Follow-up: They were followed up by outpatient visits every 3 months for the first 2 years after surgery, every 6–8 months for the next 3–5 years, and at least every 12 months thereafter. Patients who could not afford regular follow-up visits were followed up by telephone. The deadline for follow-up was December 31, 2020, and 3 cases were lost to follow-up, with a follow-up rate of 99.1%. Survival time was recorded from the date of surgery.
(7) Statistical analysis: SPSS 25.0 statistical software was employed for statistical analysis. Measurement data were analyzed by *χ*^2^ or Fisher's exact test. Categorical variables were reported as frequency and percentage. Kaplan–Meier method was used to draw survival curve, and Log-Rank test was used to assess the significance of influencing the overall survival (OS) of patients. Cox multivariate analysis and collinearity analysis were performed to explore the independent risk factors of patients' OS. Propensity score matching (PSM) method (1 : 1 matching ratio) was used for matching with the variables with different composition ratios of patients grouped according to different sites of metastatic nodes between the two groups, to eliminate the bias by balancing the observable potential confounding factors. *P *< 0.05 indicated that the difference was statistically significant.

## Results

(1) The analysis results of the composition ratio of general clinical and pathological indicators in patients with different sites of metastatic nodes

There were significant differences in the composition ratios of the three indicators, T stage, N stage and TNM stage, between the NSM group and NNSM group (*P *< 0.05) (Table 1).

(2) PSM analysis results of patients with different sites of metastatic nodes

**Table 1:** Analysis results of the composition ratio of general clinical and pathological indicators of patients before and after PSM.

Variable	Before PSM (%)		*X* ^2^	*P*	After PSM (%)		*X* ^2^	*P*
	NSM	NNSM			NSM	NNSM		
Gender			0.050	0.822			0.060	0.745
Male	90 (75.6)	155 (76.7)			77 (74.8)	79 (76.7)		
Female	29 (24.4)	47 (23.3)			26 (25.2)	24 (23.3)		
Age			1.277	0.258			0.178	0.673
<60 years	63 (52.9)	120 (59.4)			57 (55.3)	60 (58.3)		
≥60 years	56 (47.1)	82 (40.6)			46 (44.7)	43 (41.7)		
KPS			0.322	0.570			0.072	0.789
70	110 (92.4)	190 (94.1)			95 (92.2)	96 (93.2)		
≥80	9 (7.6)	12 (5.9)			8 (7.8)	7 (6.8)		
Lesion length			1.751	0.186			0.020	0.888
≤5.0 cm	69 (57.1)	100 (49.5)			58 (56.3)	57 (55.3)		
>5.0 cm	51 (42.9)	102 (50.5)			45 (43.7)	46 (44.7)		
Degree of differentiation		0.563	0.453			1.040	0.308	
Non/poorly-differentiated	24 (20.2)	34 (16.8)			19 (18.4)	25 (24.3)		
Moderately/well-differentiated	95 (79.8)	168 (83.2)			84 (81.6)	78 (75.7)		
T stage			13.891	0.000			0.036	0.849
T1 + T2	33 (27.7)	23 (11.4)			17 (16.5)	16 (15.5)		
T3 + T4	86 (72.3)	179 (88.8)			86 (83.5)	87 (84.5)		
N stage			26.565	0.000			0.033	0.856
N1	101 (84.9)	115 (56.9)			85 (82.5)	84 (81.6)		
N2 + N3	18 (15.1)	87 (43.1)			18 (17.5)	19 (18.4)		
TNM			6.006	0.014			0.116	0.733
II	9 (7.6)	4 (2.0)			5 (4.9)	4 (3.9)		
III + IVa	110 (92.4)	198 (98.0)			98 (95.1)	99 (96.1)		
Vascular tumor thrombus		0.575	0.448			0.739	0.390	
No	110 (92.4)	191 (94.6)			95 (92.2)	98 (95.1)		
Yes	9 (7.6)	11 (5.4)			8 (7.8)	5 (4.9)		
No. of lymph node dissected		0.731	0.392			0.513	0.474	
≤21	42 (35.3)	81 (40.1)			37 (35.9)	42 (40.8)		
≥22	77 (64.7)	121 (59.9)			66 (64.1)	61 (59.2)		
Postoperative adjuvant treatment		1.594	0.451			5.110	0.078	
Non-adjuvant treatment	43 (36.1)	61 (30.2)			37 (35.9)	29 (28.2)		
POCRT	33 (27.7)	55 (27.2)			30 (29.1)	22 (21.4)		
POCT	43 (36.1)	86 (42.6)			36 (35.0)	52 (50.5)		

Note: PSM, propensity score matching; KPS, Karnofsky score; PORT, postoperative radiotherapy; POCT, postoperative chemotherapy; POCRT, postoperative radiotherapy and chemotherapy; NSM, nodal skip metastasis; NNSM, no-nodal skip metastasis.

Three indicators, T stage, N stage and TNM stage were entered into the logistic binary regression model and collinearity analysis to explore the independent risk factors for NSM patients' OS. The results of collinearity analysis showed that VIF values were all less than 2 (Table 2), so collinearity between T, N and TNM stages was excluded. The results indicated that T stage and N stage were independent risk factors (*P *< 0.05) (Table 3).

**Table 2:** Collinear analysis of T, N and TNM stage.

model	Unnormalized coefficient		Normalization Coefficient	*t*	*P*	Collinear statistics	
	*B*	Error of standard	Beta			Tolerance	VIF
constant	0.940	0.422		2.226	0.027		
T stage	0.137	0.060	0.163	2.294	0.022	0.560	1.784
N stage	0.275	0.055	0.267	4.969	0.000	0.977	1.024
TNM stage	0–0.022	0.174	−0.009	−0.128	0.899	0.559	1.788

**Table 3:** Results of logistic binary regression analysis of factors affecting NSM.

Indicator	Regression coefficient	Standard error	*χ* ^2^	*P* value	OR	95% CI	
						Lower limit	Upper limit
T stage	0.916	0.348	6.910	0.009	2.499	1.262	4.948
N stage	1.366	0.298	21.059	0.000	3.921	2.187	7.027
TNM stage	0.227	0.685	0.109	0.741	1.254	0.328	4.802

With NSM as the treatment group and NNSM as the control group, and with T stage and N stage as covariates, PSM was conducted with a 1 : 1 matching ratio. The number of patients was found to be 103 in NSM group and NNSM group respectively. No significant difference was noted in the composition ratio of general clinical and pathological data after PSM between the two groups (*P *> 0.05) (Table 1).

(3) Analysis results of the effect of different sites of metastatic nodes on the prognosis of patients

**Figure 2:**
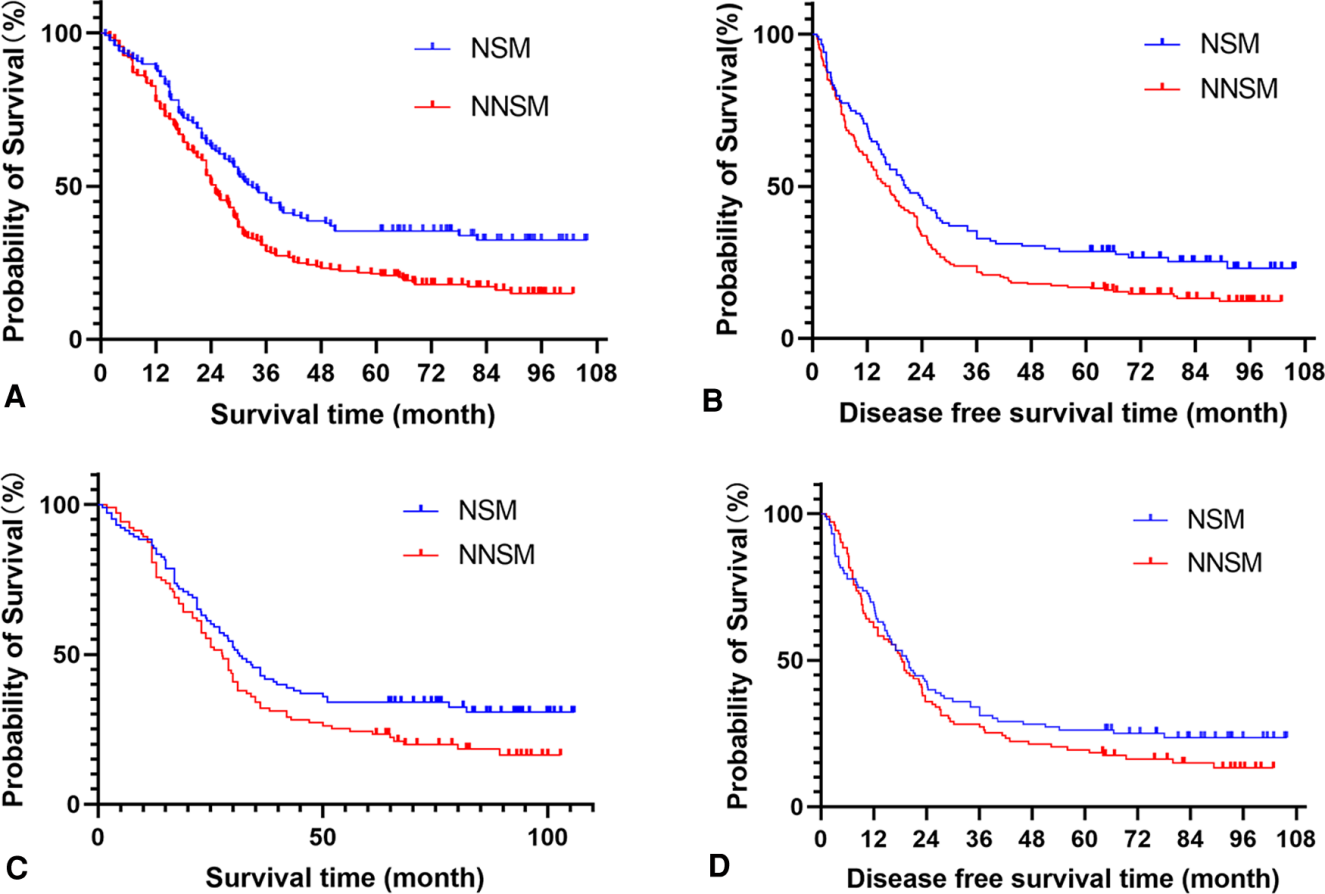
Analyse of patients survival and disease free survival time according the lymph node metastases status. (**A**) Survival analysis of all cohorts before propensity score matching. (**B**) Disease free survival analysis of all cohorts before propensity score matching. (**C**) Survival analysis of all cohorts after propensity score matching. (**D**) Disease free survival analysis of all cohorts after propensity score matching. Note: NSM: nodal skip metastasis; NNSM: no-nodal skip metastasis.

The 3- and 5-year OS rates of patients before and after PSM were 34.9%, 26.5%, and 37.4%, 29.1%, respectively, with a median OS of 27.6 months (95% CI: 24.9–30.3) and 29.0 months (95% CI: 25.8–32.2) respectively. DFS rates were 26.5%, 21.2% and 29.6%, 22.8%, respectively, with a median DFS of 18.0 months (95% CI: 15.1–20.9) and 19.0 months (95% CI: 15.9–22.1) respectively. Univariate analysis showed significant differences in OS and DFS among patients in the NSM and NNSM groups before PSM. After PSM, OS still show significant different, but no significant difference was detected in DFS after PSM (Table 4, Figure 2).

**Table 4:** Analysis results of prognosis of patients with different sites of metastatic nodes before and after PSM.

Grouping	Before PSM						After PSM					
	OS (%, month)			DFS (%, month)			OS (%, month)			DFS (%, month)		
	3-years	5-years	Median	3-years	5-years	Median	3-years	5-years	Median	3-years	5-years	Median
NSM	45.4	35.3	33.0	33.6	28.6	20.4	42.7	34.0	31.4	32.0	26.2	19.7
NNSM	28.7	21.3	25.0	22.3	16.8	16.0	32.0	24.3	27.6	27.2	19.4	18.4
*X* ^2^	10.925			6.601			3.911			1.405		
*P*	0.001			0.010			0.048			0.236		

Note: PSM, propensity score matching; NSM, nodal skip metastasis; NNSM, no-nodal skip metastasis; PSM, propensity score matching.

The potential prognostic factors of patients were entered into the COX multivariate regression model. The results showed that gender, age, N stage, site of metastatic nodes and postoperative adjuvant treatment mode were independent risk factors affecting OS of 321 patients before PSM (*P *< 0.05). Gender, age, N stage and postoperative adjuvant treatment mode were independent risk factors that affected their DFS (*P *< 0.05). Gender, N stage, site of metastatic nodes, and postoperative adjuvant treatment mode were independent risk factors for OS of 206 patients after PSM (*P *< 0.05), while gender and postoperative adjuvant treatment mode were independent risk factors that affected their DFS (*P *< 0.05) (Table 5).

(4) Analysis of different postoperative adjuvant treatment modes for patients with different sites of metastatic nodes before and after PSM

**Table 5:** Results of multivariate analysis of influencing factors of OS and DFS of patients before and after PSM.

Variable	Multivariate analysis results of influencing factors of DFS				Multivariate analysis results of influencing factors of OS			
	Before PSM (*N* = 321)		After PSM (*N* = 206)		Before PSM (*N* = 321)		After PSM (*N* = 206)	
	HR (95% CI)	*P*	HR (95% CI)	*P*	HR (95% CI)	*P*	HR (95% CI)	*P*
Gender								
Male	Reference value		Reference value		Reference value		Reference value	
Female	1.409 (1.041–1.908)	0.027	1.607 (1.087–2.378)	0.017	1.416 (1.038–1.932)	0.028	1.522 (1.012–2.290)	0.044
Age								
<60 years	Reference value		Reference value		Reference value		Reference value	
≥60 years	0.770 (0.598–0.993)	0.044	0.922 (0.671–1.268)	0.619	0.747 (0.577–0.967)	0.027	0.845 (0.610–1.172)	0.313
KPS								
70	Reference value		Reference value		Reference value		Reference value	
≥80	0.647 (0.398–1.054	0.080	0.720 (0.395–1.313)	0.284	0.703 (0.427–1.156)	0.165	0.820 (0.443–1.519)	0.529
Lesion length								
≤5.0 cm	Reference value		Reference value		Reference value		Reference value	
>5.0 cm	1.145 (0.892–1.471)	0.288	1.341 (0.976–1.843)	0.070	1.163 (0.898–1.507)	0.252	1.183 (0.0850–1.646)	0.319
Degree of differentiation								
Non/poorly-differentiated	Reference value		Reference value		Reference value		Reference value	
Moderately/well-differentiated	0.981 (0.711–1.353)	0.905	0.888 (0.603–1.307)	0.547	0.887 (0.631–1.247)	0.490	0.0748 (0.496–1.126)	0.164
T stage								
T1 + T2	Reference value		Reference value		Reference value		Reference value	
T3 + T4	0.838 (0.566–1.242)	0.380	0.667 (0.392–1.133)	0.134	0.0808 (0.539–1.213)	0.304	.0673 (0.382–1.187)	0.171
N stage								
N1	Reference value		Reference value		Reference value		Reference value	
N2 + N3	0.512 (0.391–0.670)	0.000	0.695 (0.464–1.042)	0.078	0.0481 (0.365–0.634)	0.000	0.569 (0.378–0.858)	0.007
TNM stage								
II	Reference value		Reference value		Reference value		Reference value	
III + IVa	0.643 (0.293–1.413)	0.272	0.554 (0.200–1.535)	0.256	0.655 (0.284–1.513)	0.322	.0674 (0.238–1.910)	0.457
Vascular tumor thrombus								
No	Reference value		Reference value		Reference value		Reference value	
Yes	1.091 (0.656–1.814)	0.737	1.277 (0.652–2.503)	0.476	1.126 (0.648–1.958)	0.674	1.004 (0.493–2.046)	0.991
No. of lymph node dissected								
≤21	Reference value		Reference value		Reference value		Reference value	
≥22	1.116 (0.865–1.440)	0.399	0.996 (0.720–1.376)	0.979	1.134 (0.872–1.475)	0.349	1.251 (0.891–1.758)	0.196
Site of metastatic nodes								
NSM	Reference value		Reference value		Reference value		Reference value	
NNSM	0.818 (0.626–1.070)	0.143	0.837 (0.610–1.151)	0.274	0.749 (0.565–0.0994)	0.045	0.700 (0.505–0.971)	0.033
Postoperative adjuvant treatment								
Non-adjuvant treatment	Reference value		Reference value		Reference value		Reference value	
POCRT	0.937 (0.703–1.248)	0.656	0.922 (0.644–1.318)	0.656	1.253 (0.935–1.678)	0.131	1.225 (0.850–1.765)	0.277
POCT	0.584 (0.424–0.805)	0.001	0.509 (0.333–0.777)	0.002	0.648 (0.465–0.901)	0.010	0.0580 (0.373–0.900)	0.015

Note: PSM, propensity score matching; NSM, nodal skip metastasis; NNSM, no-nodal skip metastasis; PORT, postoperative radiotherapy; POCT, postoperative chemotherapy; POCRT, postoperative radiotherapy and chemotherapy.

**Figure 3:**
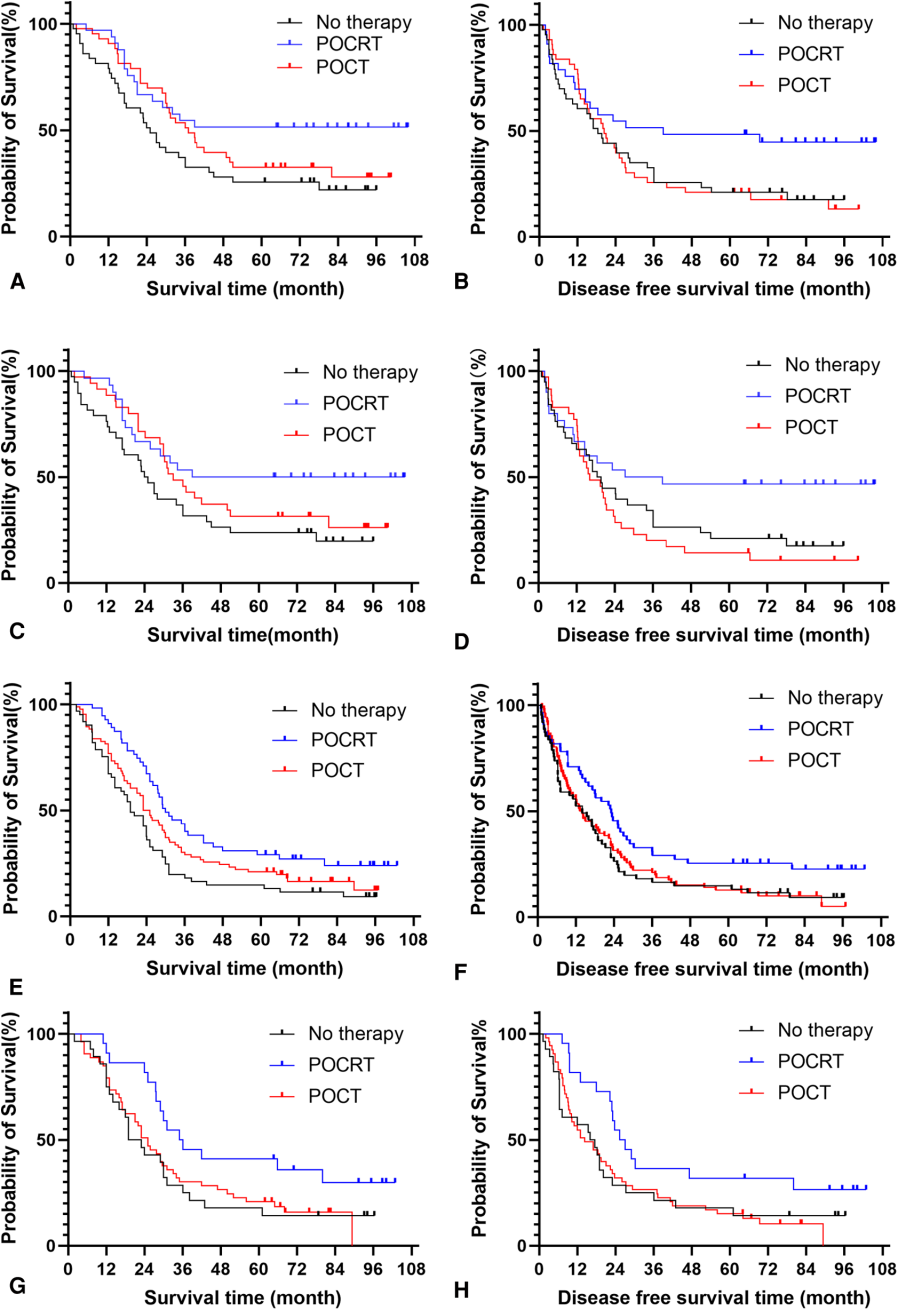
Analysis of postoperative adjuvant treatment modes for patients with different sites of metastatic nodes before and after propensity score matching. (**A**) Survival analysis of NSM patients before propensity score matching. (**B**) Disease free survival analysis of NSM patients before propensity score matching. (**C**) Survival analysis of NSM patients after propensity score matching. (**D**) Disease free survival analysis of NSM patients after propensity score matching. (**E**) Survival analysis of NNSM patients before propensity score matching. (**F**) Disease free survival analysis of NNSM patients before propensity score matching. (**G**) Survival analysis of NNSM patients after propensity score matching. (**H**) Disease free survival analysis of NNSM patients after propensity score matching. Note: NSM: nodal skip metastasis; NNSM: no-nodal skip metastasis; PORT: postoperative radiotherapy; POCT: postoperative chemotherapy; POCRT: postoperative radiotherapy and chemotherapy.

The results showed that POCRT showed good efficacy in 321 patients before PSM regardless of their metastatic nodes status (*P *< 0.05) (Table 6). POCRT also had significant clinical benefits including prolongation of OS and DFS of patients in the NSM group after PSM (*P *< 0.05), while patients in the NNSM group tended to gain survival benefit from postoperative adjuvant treatment,though OS and DFS did not reacht significant different (*P *> 0.05) (Table 7, Figure 3).

(5) Failure mode

**Table 6:** Effect of different postoperative adjuvant treatment modes on the prognosis of patients with different sites of metastatic nodes before PSM.

Treatment mode	*N*	OS (%, month)			*X* ^2^	*P*	DFS (%, month)			*X* ^2^	*P*
		3-years	5-years	median			3-years	5-years	median		
NSM					6.711	0.035				5.651	0.049
Non-adjuvant treatment	43	32.6	25.6	25.0			27.9	20.9	18.5		
POCRT	33	54.5	51.5	–			51.5	48.5	39.0		
POCT	43	51.2	32.6	37.0			25.6	20.9	20.4		
NNSM					9.893	0.007				6.413	0.040
Non-adjuvant treatment	61	18.0	14.8	19.0			16.4	14.8	14.0		
POCRT	55	40.0	29.1	30.0			30.9	25.5	23.1		
POCT	86	29.1	20.9	23.0			20.9	12.8	13.3		

NSM, nodal skip metastasis; NNSM, no-nodal skip metastasis; PSM, propensity score matching; PORT, postoperative radiotherapy; POCT, postoperative chemotherapy; POCRT, postoperative radiotherapy and chemotherapy.

**Table 7:** Effect of different postoperative adjuvant treatment modes on the prognosis of patients with different sites of metastatic nodes after PSM.

Treatment mode	*N*	OS (%, month)			*X* ^2^	*P*	DFS (%, month)			*X* ^2^	*P*
		3-years	5-years	median			3-years	5-years	median		
**NSM**					6.188	0.045				6.257	0.041
Non-adjuvant treatment	38	31.6	26.3	23.9			34.2	21.1	18.5		
POCRT	30	53.3	50.3	39.0			50.0	46.7	27.3		
POCT	35	45.7	31.4	33.0			20.0	14.3	16.1		
**NNSM**					5.164	0.076				5.453	0.065
Non-adjuvant treatment	28	25.0	17.9	19.0			21.4	17.9	16.0		
POCRT	22	45.5	40.9	35.0			36.4	31.8	25.3		
POCT	53	30.2	20.8	25.0			26.4	15.1	14.3		

NSM, nodal skip metastasis; NNSM, no-nodal skip metastasis; PORT, postoperative radiotherapy; POCT, postoperative chemotherapy; POCRT, postoperative radiotherapy and chemotherapy.

Of 321 patients, 146 cases (45.5%) had local regional recurrence, 104 cases (32.4%) had distant metastasis, and 49 cases (15.3%) had local recurrence and distant metastasis until the date of last follow-up. There were 57 (47.9%) and 89 (44.1%) patients with recurrence in the NSM and NNSM groups, respectively, suggesting no significant difference between the two (*X*^2 ^= 0.445, *P *= 0.505), in addition to (30.3%) and 68 (33.7%) with distant metastases respectively, suggesting no significant difference (*X*^2 ^= 0.398, *P *= 0.528).

After PSM, among the 206 patients, 95 cases (46.1%) had local regional recurrence, 65 cases (31.6%) had distant metastasis, and 31 cases (15.0%) had local regional recurrence and distant metastasis. There were 49 (47.6%) and 46 (44.7%) patients with recurrence in the NSM and NNSM groups, respectively, suggesting no significant difference (*X*^2 ^= 0.176, *P *= 0.675); distant metastases were found in 31 patients (30.1%) and 34 cases (33.0%) respectively, suggesting no significant difference (*X*^2 ^= 0.202, *P *= 0.653).

## Discussion

Lymph node metastasis is one of the poor prognostic indicators for patients with esophageal cancer after esophagectomy. The number of positive lymph nodes has been included in N stage by AJCC staging system for esophageal cancer, and is widely recognized and applied by clinicians as an important prognostic factor for patients undergoing esophagectomy (14). However, another important prognostic factor, the distribution of positive lymph nodes, has not been included, but valued by the JES TNM staging for esophageal cancer (13). In clinical practice, the significance of site of positive metastatic nodes in the prognosis of esophageal cancer patients has not attracted sufficient attention. This is related to the insufficient clinical research on site of metastatic nodes in esophageal cancer patients and the inconsistent research conclusions. In this study, we analyzed the mid-thoracic esophageal cancer which had the highest incidence in esophageal cancer and the highest incidence rate of NSM. A retrospective analysis of 321 MT-ESCC patients in this study suggested that patients with different sites of metastatic nodes had different prognosis. Among them, the prognosis of patients in the NSM group was significantly better than that of the NNSM group, and the site of metastatic nodes was one of the independent risk factors for predicting the prognosis of patients. This was similar to the results of Xu et al. (8) in which the clinical data of 300 MT-ESCC patients with lymph node metastasis were retrospectively studied, including 66 cases (22.0%) in the NSM group. The study showed that the prognosis of patients before and after PSM was better in the NSM group than in the NNSM group (Before PSM, 3-year OS was 62.1% vs. 34.1%, *P *< 0.001; after PSM, 3-year OS was 66.7% vs. 40.0%, *P *= 0.025). Subsequent multivariate analysis revealed that NSM was independent factors responsible for OS benefit in MT-ESCC patients. However, several studies reported that NSM cannot be used to predict the prognosis of patients undergoing esophagectomy. For example, Zhu et al. (15) retrospectively analyzed 207 patients undergoing esophagectomy for esophageal cancer, including 58 patients (26%) who developed NSM. The median OS of those patients was 30 months, and the 3- and 5-year OS rates were 42.3% and 36.7%, respectively. NSM was not a significant prognostic factor for all patients in the whole group or those with mid-thoracic esophageal cancer (*n* = 131) (*P *= 0.767, 0.864). The results of this study showed that NSM was a significant prognostic factor for prolongation of OS and DFS in the NSM group before and after PSM, as compared with NNSM group, but DFS did not show a significant survival benefit after PSM in the NSM group. This might be related to the small proportion of patients in NSM group at the earlier T stage and N stage after PSM.

Given that the mechanism of lymph node metastasis may be related to the lymphatic drainage, micrometastasis and the biological behavior of tumor cells at the anatomical location of the tumor, the favorable prognosis of NSM patients may be related to the following factors. First, in this study, the grouping of 321 patients before PSM showed that the proportion of patients at the earlier T stage and N stage was higher in the NSM group than in the NNSM group, which was similar to many previous related studies (4, 8, 9, 16–18). Second, the number of sites of metastatic nodes also affected the prognosis of patients undergoing surgery for esophageal cancer (19). In this study, lymph node metastasis was detected in merely one region in the NSM group was one, while 42.6% (86/202) of patients in the NNSM group had lymph node metastasis in 2–3 regions. Third, tumor biological factors were involved in the occurrence and development of NSM, and the biological characteristics of tumors might play an essential role in the skip metastasis in tumor cells, leading to relatively lower malignancy of tumor cells in NSM patients, thereby contributing to better prognosis (20, 21).

Neoadjuvant chemoradiotherapy is currently the recommended treatment plan for patients with locally advanced esophageal cancer. However, due to various reasons, most centers in China have provided postoperative adjuvant treatment, especially in the adjuvant treatment mode of esophageal cancer in the past few years. Due to the lack of robust evidence from the perspective of evidence-based medicine, postoperative adjuvant therapy of esophageal cancer has currently not been recommended in relevant treatment guidelines for esophageal cancer. However, it has been widely accepted by most clinicians that postoperative adjuvant treatment provides survival benefit for patients with lymph node-positive esophageal cancer. However, the individuals who do gain benefit from adjuvant therapy after esophageal cancer surgery need to be further explored. In this study, we analyzed the efficacy and prognosis of different postoperative adjuvant treatment options for patients with different sites of metastatic nodes. The results showed that POCRT promoted survival benefits, specifically prolongation of OS, in the NSM group. Among 2,285 patients undergoing surgery for esophageal cancer in a study, Shang et al. (4) divided 1,137 with lymph node metastasis into NSM group (*n* = 156), local lymph node metastasis (LNM) group (*n* = 665) and mediastinal lymph node metastasis (MNM) group (*n* = 316). Several patients received adjuvant POCRT. The results suggested that the prognosis of patients was significantly better in the LNM group than in the MNM group after adjuvant therapy (*P *< 0.05), but significantly worse in the LNM group than in the NSM group, showing no significant difference (*P *> 0.05). This study was different from our study, in which the patients were grouped in a Chinese fashion. Specifically, the former had subdivision of NSM, while the latter did not make subdivision of local lymph nodes and non-local lymph nodes. In this study, postoperative adjuvant treatment did not provide benefit in DFS of patients in the NNSM group after PSM. One of the reasons might be that there were >2 regions of lymph node metastasis in the NNSM group, which led to poor inherence in the prognosis in this group. The second reason is that in this retrospective study, the postoperative adjuvant treatment schemes were diverse for patients, which might affect the efficacy. This study had a small sample size, which might also affect the results of the study to a certain extent. In addition, other studies on postoperative adjuvant therapy have also confirmed that postoperative adjuvant treatment could improve the patient's OS, but could not increase DFS significantly (12).

This study has several limitations. First, this study is a retrospective study, which is not as convincing as a prospective study. Second, the conclusions of this single-center study cannot be generalized to all diagnosis and treatment centers for esophageal cancer. Third, to elucidate the predictive effect of NSM on patients undergoing esophageal cancer surgery, only patients with mid-thoracic esophageal cancer were enrolled in this study, and the remaining esophageal sites were excluded. This may have introduced inevitable selection bias. Fourth, a small sample size in this study may affect the results of the study to a certain extent. In addition, some patients in this study received Sweet surgery, which led to limited scope of neck lymph node dissection. However, PSM analysis was performed in this study to minimize the bias by balancing the potential confounding factors in the study. Moreover, in this study, we found that these patients with NSM after esophageal cancer surgery might have special prognosis. An issue that should arouse great concern is whether the current defining of N stage in the postoperative TNM staging of esophageal cancer with the number of lymph nodes is applicable in clinical practice? In addition, patients with lymph node metastasis may receive different postoperative adjuvant treatments, and the survival benefits from postoperative adjuvant treatments may vary from person to person. All these need further exploration and in-depth study in clinical practice.

In conclusion, NSM is a good prognostic factor for patients receiving surgery for MT-ESCC. It is recommended that MT-ESCC patients with NSM after esophagectomy undergo postoperative adjuvant chemoradiotherapy, thereby gaining survival benefits,. The final conclusion needs to be confirmed by multi-center, prospective, randomized controlled studies.

## Data Availability

The raw data supporting the conclusions of this article will be made available by the authors, without undue reservation.
